# Risk of Cervical Spine Complications in Individuals with Cervical Dystonia Treated with Botulinum Toxin Therapy

**DOI:** 10.5334/tohm.1203

**Published:** 2026-05-20

**Authors:** Samyukta Senthil, Sanjeev Kumar, Aparna Wagle Shukla

**Affiliations:** 1 University of Florida, Gainesville, Florida, United States of America; 2 Department of Anesthesiology/Pain Medicine, University of Florida, Gainesville, Florida, United States of America; 3 Department of Neurology, Fixel Institute for Neurological Diseases, University of Florida, Gainesville, Florida, United States of America

**Keywords:** cervical dystonia, cervical spine stenosis, myelopathy, radiculopathy, botulinum toxin, medial branch block

## Abstract

**Objective:**

To examine the risk of cervical spine complications in individuals with focal cervical dystonia (CD) treated with botulinum toxin (BoNT) injections.

**Background:**

CD is a movement disorder characterized by involuntary twisting and turning of the neck, often accompanied by a jerky tremor. Owing to the persistent abnormal forces exerted on the cervical spine, cervical spine pathology has been reported in approximately 30% of patients; however, data on this risk in the context of BoNT therapy are lacking.

**Design/Methods:**

In a retrospective analysis of CD patients receiving BoNT therapy, we determined the prevalence of cervical spine complications, including degenerative cervical spinal stenosis, myelopathy, radiculopathy, and atlantoaxial dislocation. Data on treatments including pain medications and interventions both surgical such as cervical laminectomy, discectomy, anterior cervical discectomy and fusion (ACDF), posterior cervical instrumentation and fusion (PCF), and non-surgical like radiofrequency ablation, and medial branch blocks were extracted. In addition, we conducted a literature review of cervical spine complications reported as associated with CD.

**Results:**

In a cohort of 320 patients receiving regular BoNT therapy and followed longitudinally for 5 or more years, we found 17 individuals (5.3%) with new onset cervical stenosis (n = 11), radiculopathy (n = 5) or myelopathy (n = 4), developing after the diagnosis of CD and initiation of BoNT therapy (58.8% developing in the first 5 years). The neck pain in these individuals was managed with opioid medications in addition to BoNT injections and oral muscle relaxants. While procedures such as medial branch blocks (n = 5), radiofrequency ablation (n = 2), and epidural steroid injections (n = 8) were employed for controlling pain, surgical interventions such as laminectomy (n = 3), discectomy (n = 4), PCF (n = 1), and ACDF (n = 3) were warranted in some individuals.

**Conclusion:**

Cervical spine complications following a diagnosis of CD may necessitate opioid therapy, nerve blocks, ablative procedures, and/or surgical interventions. In contrast to prior reports from the predominantly pre–BoNT era, which estimated complication rates of approximately 30%, our cohort, well managed with BoNT therapy, demonstrated a considerably lower risk of cervical spine complications (5.3%). These findings suggest that early initiation of BoNT therapy, by effectively controlling abnormal involuntary forces exerted on the cervical spine, may reduce the risk of secondary cervical spine complications.

## Introduction/Background

Isolated focal cervical dystonia (CD) is a neurological disorder characterized by involuntary twisting and turning of the neck muscles. As a lifelong condition in most patients with no known cure, it often leads to chronic pain and significantly impacts the quality of life [1,2]. Cervical spondylosis refers to the progressive degeneration of the bones and discs in the cervical spine. It is commonly associated with aging and can lead to symptoms such as neck pain, stiffness, and reduced mobility [3]. Some studies have found that there is increased prevalence of severe degenerative disease of the upper cervical spine such as cervical myelopathy, cervical radiculopathy, atlantoaxial dislocation or subluxation in the presence of a diagnosis of CD [4,5,6]. These studies suggest that such sequela may be a result of persistent involuntary forces exerted on the spine, as observed in CD [5]. From a clinical standpoint, patients with a dual diagnosis of CD and cervical spine disease are likely to experience an increased symptom burden and may report persistent symptoms despite receiving adequate doses of standard treatments for CD. These patients may require procedures such as medial branch block, epidural injections and radiofrequency ablation for addressing pain arising from the cervical facet joint or nerve root/s impingement. In some cases, surgical interventions such as cervical laminectomy, discectomy, anterior cervical discectomy and fusion (ACDF) and posterior cervical instrumented fusion (PCF) may be needed to alleviate symptoms and improve spinal stability particularly when conservative pain management proves ineffective [5].

Although there are reports linking CD to cervical spine complications, to date, most published data has lacked detailed information on the timeline for the onset of cervical spine complications following a diagnosis of CD and the potential impact of botulinum toxin (BoNT) treatment, first line therapy when introduced early in altering the trajectory of clinical experiences. In this study, we sought to examine the following questions: (1) Determine the prevalence of significant cervical spine disease in CD patients receiving regular BoNT therapy. (2) Evaluate the patterns of pharmacological treatments such as use of opioid medications, (in addition to BoNT), pain control procedures, and surgical interventions. (3) Review the literature on the prevalence and extent of cervical spine complications occurring in individuals diagnosed with isolated CD and compare the findings with those identified in our cohort.

## Methods

Upon receiving approval from the local institutional review board (IRB202300096) at the University of Florida, we reviewed the electronic health records of patients followed at our movement disorders center and found 320 patients who had received longitudinal (≥5 years) EMG guided BoNT therapy at our clinic. We then considered the following inclusion criteria for selection (1) CD diagnosis established at UF by a movement disorder neurologist and followed in our BoNT injection clinic for a minimum of 5 years. (2) Longitudinal data available in our EMR (Epic) regarding the development of significant cervical spine disease such as cervical stenosis, disc prolapse, radiculopathy, myelopathy, fractures or atlantoaxial dislocation following the diagnosis of CD. These conditions necessitated consideration for surgical or interventional pain management procedures. Although some patients may have received treatment for spinal complications at outside facilities, only those who continued BoNT treatment at our clinic before and after procedures were included. Subjects were excluded from the final cohort if the timeline of spinal complications or related procedures was unclear or unavailable.

We extracted data regarding demographics, CD related features, timeline for development of significant cervical spine disease, and the spine level affected (whether upper cervical spine that is more mobile or lower cervical spine that is less mobile). We extracted data regarding the BoNT injections (formulation, doses, timing of introduction in the clinical course and treatment response). To analyze the symptomatic benefits of the BoNT, we asked the patients to rank their improvement on a subjective scale from 0–100%. No improvement was outlined as 0%, mild improvement as 1–25%, moderate improvement as 26–50%, marked improvement as 51–75%, and complete improvement was described as 76–100% improvement. We obtained data on concomitant oral medication therapy used for treating CD specifically the prescription of muscle relaxants (cyclobenzaprine, methocarbamol, chlorzoxazone), GABAergic drugs such as benzodiazepines (clonazepam, diazepam, lorazepam, alprazolam) or baclofen, anticholinergic drugs (benztropine, trihexyphenidyl) and dopaminergic drugs. We extracted data on pain control medications including gabapentin, pregabalin, SSRIs or SNRIs (duloxetine, venlafaxine, citalopram, escitalopram) and opioids/scheduled substances (hydrocodone, oxycodone, tramadol, and/or morphine). We extracted data on nonsurgical interventions such as epidural steroid injections, nerve block procedures (specifically medial branch block) and radiofrequency ablation of cervical spine nerves for management of cervical spine symptoms. We extracted data on surgical interventions such as laminectomy, discectomy, ACDF, or PCF instrumentation performed in these individuals. We examined the number of individuals requiring multiple procedures for controlling the spinal symptoms. For our third aim, we searched the PubMed database using the terms (“cervical dystonia” [Mesh] OR “cervical dystonia” [Title/Abstract]) AND (“cervical spine disease” [Mesh] OR “cervical spine disease” [Title/Abstract]). Filters included articles written in English and studies conducted on human subjects. We excluded studies with data on cerebral palsy, congenital muscular dystrophy, complex genetic dystonia, and if CD followed the diagnosis of cervical spine disease.

## Results

In a cohort of 320 CD patients followed longitudinally for at least 5 years in our BoNT practice, we identified 17 individuals (5.3%) who developed significant cervical spine disease after the establishment of CD diagnosis and initiation of BoNT therapy. There were 6 males and 11 females with mean (± SD) age of 68.9 ± 13.9 years and mean disease duration of 12 ± 5 years. The median latency to diagnosis of CD and/or BoNT initiation from onset of symptoms was 3 years (range 1 to 8). Diagnosis was idiopathic in 76.5% and related to post-traumatic brain injury in 23.5%. No family history of dystonia was present in any case. There were 12 patients (70.6%) with a concurrent diagnosis of dystonic head tremor, 2 patients (11.8%) at the time of chart review had segmental spread of dystonia symptoms to arms, jaw, or eyes.

**Figure 1:**
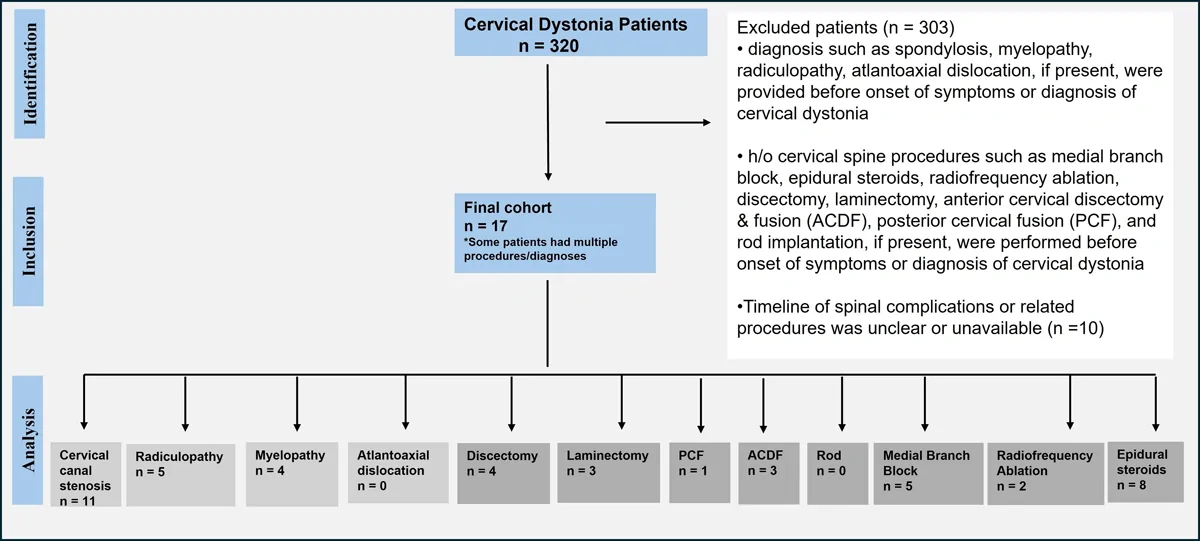
Flowchart of included and excluded patients.

All 17 patients had initiated BoNT injections immediately after diagnosis and received injections every 12 weeks for the length of follow-up (mean length of follow-up, 12.5 ± 3.9 years). These patients endorsed the following response pattern; 29.4% of subjects reported ‘mild improvement,’ 52.9% reported ‘moderate improvement,’ 17.6% indicated ‘marked improvement.’ 11 patients had a diagnosis of cervical stenosis, 5 patients had radiculopathy, 4 patients with myelopathy. There was no patient identified with a diagnosis of atlantoaxial dislocation. 11 patients developed cervical spine disease in C2/C3/C4 level. 10 patients were noted to develop cervical spine disease within 5 years of diagnosis confirmation (immediately followed by initiation of BoNT), 4 patients developed 5–10 years later, and 3 patients >10 years after the diagnosis of CD. Figure 1 shows the number of participants that were included and excluded. Table 1 details the individual subject data.

**Table 1:** Demographics and Clinical characteristics.


S	AGE IN YRS	SEX	YRS SINCE CD ONSET	YRS SINCE CD DX	INTERVAL BETWEEN CD ONSET & CERVICAL SPINE DX IN YRS	CERVICAL SPINE DIAGNOSIS				SURGICAL PROCEDURES					NON–SURGICAL PAIN PROCEDURES			CERVICAL SPINE LEVEL	MULTIPLE SPINE PROCEDURES
		
						CCS	RADI	MYEL	AAD	DISC	PCF	ACDF	LAMI	RODS	MBB	RA	EPIDURAL STEROID &/OR LIDOCAINE		

1	59	F	13	9	<5	+	+	+	–	+	–	+	+	–	–	+	+	C4, C5, C6	+

2	76	F	18	15	5–10	–	–	–	–	+	–	+	+	–	+	–	+	C2, C3, C4, C5, C6, C7	+

3	65	F	15	15	5	+	–	+	–	+	+	+	+	–	–	–	–	C4, C5	+

4	64	F	6	6	<5	+	+	–	–	+	–	–	–	–	+	–	–	C2, C3, C4, C5	+

5	65	M	21	14	<5	–	+	+	–	–	–	–	–	–	–	–	–	C4, C5, C6	+

6	37	M	17	2	<5	–	–	–	–	–	–	–	–	–	+	+	+	C2, C3	+

7	83	F	14	14	<5	+	–	–	–	–	–	–	–	–	–	–	–	C3, C4, C5, C6	–

8	46	M	6	5	<5	+	–	+	–	–	–	–	–	–	–	–	+	C3, C4	–

9	90	M	24	21	>10	–	+	–	–	–	–	–	–	–	–	–	–	unknown	–

10	79	M	15	15	5–10	+	–	–	–	–	–	–	–	–	–	–	+	C5, C6, C7	–

11	71	F	21	17	>10	+	–	–	–	–	–	–	–	–	–	–	+	unknown	–

12	55	F	6	6	<5	+	–	–	–	–	–	–	–	–	+	–	–	C4, C5, C6	–

13	76	F	16	16	5–10	–	–	–	–	–	–	–	–	–	+	–	–	unknown	–

14	68	F	8	8	<5	+	–	–	–	–	–	–	–	–	–	–	–	unknown	–

15	85	F	8	8	<5	+	–	–	–	–	–	–	–	–	–	–	+	C4, C5, C6	–

16	76	M	11	10	5–10	+	–	–	–	–	–	–	–	–	–	–	+	C4, C5, C6	–

17	76	F	17	17	>10	–	+	–	–	–	–	–	–	–	–	–	–	C5, C6, C7, C8	–


+, present; –, absent; Subject number, S; cervical canal stenosis, CCS; radiculopathy, Radi; myelopathy, Myel; atlantoaxial dislocation, AAD; discectomy, Disc; posterior cervical fusion, PCF; anterior cervical discectomy and fusion, ACDF; laminectomy, Lami; medial branch block, MBB; radiofrequency ablation, RA.

All 17 patients were prescribed opioids following the diagnosis of the cervical spine disease. 11 patients (64.7%) used oxycodone (mean dose 40–50 mg/day), 5 patients (29.4%) used hydrocodone (mean dose 30 mg/day). Some patients needed to be switched to morphine or fentanyl (mean dose 20 mg/day and 33 mg/day respectively) or tramadol (mean dose 200 mg/day). Muscle relaxants and benzodiazepines were the other most common concomitant oral therapies. There were 16 patients on muscle relaxants: 12 on cyclobenzaprine, 2 on methocarbamol, and 2 using a combination of muscle relaxants. 14 patients were noted to have been treated with at least one of the benzodiazepines at some point after the diagnosis of CD. Among these, 9 used clonazepam, 7 used diazepam, 6 used lorazepam, and 4 used alprazolam. There were 6 patients on trihexyphenidyl, and no patients used benztropine Then there were 12 patients using gabapentin, 3 using pregabalin, 8 using venlafaxine, and 5 using duloxetine in addition to opioids, muscle relaxants and benzodiazepines. (Table 2).

**Table 2:** Pharmacological treatments for cervical dystonia and cervical spine diagnosis.


S	MUSCLE RELAXANTS: CHZ, CYCLO, MCB, MTX	BENZODIAZEPINES: CLZ, DZP, LZP, ALP	ANTICHOLINERGICS: BZT, THP	OTHER PSYCHOTROPIC MEDS: GBP, PGB, DUL, VEN	PAIN MEDICATIONS: HC, OXY TRD, MS, FEN	BOTULINUM (BONT) FORMULATION AND DOSE	IMPROVEMENT RESPONSE TO BOTULINUM AFTER CERVICAL SPINE DIAGNOSIS

1	CYCLO (5 mg), MCB (1000 mg)	CLZ* (1 mg), DZP (5 mg)	THP (4 mg)	GBP (600 mg), PGB* (100 mg), VEN* (75 mg)	HC (5–325 mg), TRD (50 mg), OXY (10 mg), FEN (2 mg)	Ona-BoNT; 300 units	moderate

2	CYCLO (15 mg)	LZP (1 mg), ALP (0.5 mg)	THP (6 mg)	GBP (1800 mg), PGB (100 mg), DUL (30 mg), VEN (37.5 mg)	OXY (10 mg)	Rima-BoNT; 10000 units	moderate

3	CYCLO (30 mg), MCB (2250 mg)	CLZ* (2 mg), DZP (5 mg), ALP (1 mg)	THP (4 mg)	GBP (300 mg), PGB* (450 mg)	OXY (5 mg), FEN (25 mcg), HC (10 mg)	Rima-BoNT; 5000 units	moderate

4	CYCLO (30 mg)	DZP (15 mg), LZP* (0.5 mg)	N/A	GBP (no dosage specified)	HC (5–325 mg), OXY (10–325 mg), MS (4 mg)	Ona-BoNT; 400 units	mild

5	N/A	N/A	THP* (6 mg)	GBP (600 mg)	TRD (50 mg)	Ona-BoNT; 100 units	moderate

6	CYCLO (10 mg)	CLZ* (1 mg)	N/A	N/A	FEN (50–250 mcg)	Ona-BoNT; 400 units	mild

7	CYCLO (5 mg)	CLZ* (0.5 mg), LZP (1 mg), ALP (0.5 mg)	N/A	GBP (1200 mg)	TRD (50 mg), OXY (5 mg),	Ona-BoNT; 300 units	marked

8	CYCLO (30 mg)	DZP (10 mg), LZP* (1 mg)	N/A	GBP* (300 mg)	OXY (10 mg), TRD (50 mg), MS (15 mg)	Ona-BoNT; 400 units	moderate

9	CYCLO (15 mg)	CLZ* (1.5 mg)	N/A	DUL (30 mg)	TRD (100 mg)	Ona-BoNT; 360 units	mild

10	CYCLO (15 mg)	CLZ* (3 mg)	THP (4 mg)	VEN (75 mg)	OXY (10 mg), TRD (50 mg), MS (15 mg), FEN (50 mcg)	Inco-BoNT; 350 units	mild

11	MCB (500 mg)	CLZ* (1.5 mg), DZP (5 mg)	N/A	GBP (300 mg), VEN (75 mg)	OXY (10 mg), TRD (50 mg), MS (50 mcg)	Rima-BoNT; 15000 units	moderate

12	CYCLO (5 mg)	LZP* (1 mg), DZP (10 mg)	N/A	GBP* (1800 mg), DUL (120 mg), VEN (75 mg)	OXY (10 mg), MS (30 mg), HC (5–325 mg), TRD (50 mg)	Ona-BoNT; 300 units	mild

13	CYCLO* (15 mg)	CLZ (1 mg)	N/A	N/A	*BACC	Rima-BoNT; 15000 units	moderate

14	CYCLO (15 mg)	CLZ (1 mg)	THP (4 mg)	DUL (20 mg), VEN (37.5)	TRD (50 mg), MS (2 mg)	Ona-BoNT; 100 units	moderate

15	CYCLO (15 mg)	DZP (5 mg), LZP (2 mg), ALP (0.25 mg)	N/A	GBP (200 mg), VEN (75 mg)	HC (5–325 mg), OXY (5–325 mg), TRD (50 mg), MS (2 mg)	Ona-BoNT; 240 units	moderate

16	CYCLO* (30 mg)	N/A	N/A	GBP* (300 mg), DUL* (60 mg), VEN* (75 mg)	TRD (50 mg), MS (2 mg)	Ona-BoNT; 450 units	moderate

17	MCB* (750 mg)	N/A	N/A	GBP* (600 mg)	OXY (50 mg), TRD (50 mg)	Ona-BoNT; 200 units	marked


Subject number, S; chlorzoxazone, CHZ; Cyclobenzaprine, CYCLO; methocarbamol, MCB; metaxalone, MTX; clonazepam, CLZ; diazepam, DZP; lorazepam, LZP; alprazolam, ALP; benztropine, BZT; trihexyphenidyl, THP; hydrocodone, HYD; Oxycodone, Oxy; tramadol, TML; morphine, MOR; Fentanyl, Fent; gabapentin, GBP; pregabalin, PGB; duloxetine, DUL; venlafaxine, VEN; Botulinum toxin, BoNT; Ona botulinum toxin type A, Ona; Inco botulinum toxin type A, Inco; Rima botulinum toxin type B, Rima; butalbital-acetaminophen-caffeine-codeine, BACC. * Active meds.

11 patients (64.7%) were treated with non-surgical pain procedures. Among these 5 patients with medial branch block, 8 patients with epidural steroid injections (and/or lidocaine) and 2 patients with radiofrequency ablation. Seven patients (41%) required more than one procedure for control of symptoms. 4 patients (23.5%) required surgical interventions, 3 were treated with ACDF, 3 with laminectomy, 4 with discectomy, one patient needed PCF (Table 2).

Table 3 summarizes the literature on cervical spine complications in the context of CD [4,6,7,8,9,10,11,12,13,14,15,16,17]. As many of the reports were case reports (n = 5) or case series (n = 5), the prevalence rates could not be determined. In some reports it was not clear whether cervical spine disease occurred consequently or coexisted as a comorbid condition. Two of the case series published by Wong et al. and Adler et al. included CD among multiple diagnoses in their case series. Clinical details specifically applicable to CD were extracted from these publications. The prevalence rate could be determined for three retrospective chart reviews published by Jankovic et al. [4] in 1991 (32%), Chawda et al. [13] in 2000 (41%), and Hagenah et al. [10] in 2001 (18%). Compared with our patient cohort that was older in age, analysis of the published reports with available data (13 publications) showed a mean age of 44.5 years, with a predominance of male patients. The mean age at onset of CD was 36.5 years (9 publications) and three reports described cases of childhood-onset dystonia. Geographically, four publications originated from North America, six from Europe, and three from Asia. Notably, 10 of 13 reports documented involvement of the upper cervical spine (C1–C3). Myelopathy was reported in nine reports, while four involved disc fusion.

**Table 3:** Summary of Study Characteristics from Published Literature (chronologically).


AUTHOR	STUDY TYPE	YEAR OF PUBLICATION	COUNTRY, CONTINENT	CERVICAL SPINE DIAGNOSIS	N/N	MEAN AGE IN YEARS	WHAT % DEVELOP	M:F	AGE OF ONSET

Chen et al. REF 6	Case series	2012	Taiwan, Asia	cervical myelopathy, cord compression at C3–C4, C4–C5, & C5–C6, discectomy & interbody fusion cage at C3–C4	10/10	53	N/A	7:3	24.9

Wong et al. REF 7	Case series	2005	Canada, North America	cervical myeloradiculopathy, disc fusion C1–C2 laminectomy C4–C5	1/8	44	N/A	1:0	44

Krauss et al. REF 8	Case series	2002	Germany, Europe	cervical myelopathy, cervical spondylosis at C3–C4 & C4–C5, anterior plate stabilization C3–C5	8/8	43	N/A	5:3	34

Hagenah et al. REF 9	Chart review	2001	Germany, Europe	cervical radiculopathy and myelopathy, spondylosis at C2–C3, C4–C5, & C5–C6, sequestrectomy, herniation of C6–C7	6/34	53.6	18%	3:3	45.9

Weigel et al. REF 10	Case report	2001	Germany, Europe	osseous fusion, partial myectomy, posterior ramisectomy C1–C6	1/1	31	N/A	1:0	23

Dalvie et al. REF 13	Case report	2000	India, Asia	C1–C2 rotary subluxation, posterior fusion of C1–C2	1/1	37	N/A	1:0	N/A

Al–Jishi et al. REF 11	Case report	2000	Bahrain, Asia	atlantoaxial subluxation & cord compression at C5–C6 & C6–C7, cervical myeloradiculopathy, disc fusion	1/1	46	N/A	1:0	“childhood”

Chawda et al. REF 12	Case series	2000	United Kingdom, Europe	cervical spondylosis at C2–C3 & C3–C4, selective peripheral denervation surgery	14/34	53.5	41%	6:8	42

Adler et al. REF 14	Case series	1996	USA, North America	cervical radiculomyelopathy, C5 fracture, anterior fusion at C6–C7, posterior fusion, laminectomy at C3–T2, post operative botulinum toxin	3/4	N/A	N/A	1:0	N/A

Defazio et al. REF 15	Case report	1993	Italy, Europe	cervical radiculopathy, cervical root impairment at C7–T1	1/1	49	N/A	0:1	“childhood”

Polk et al. REF 16	Case report	1992	USA, North America	cervical radiculopathy & myelopathy, spondylosis at C3–C7, cord compression, laminectomy C3–C7	1/1	34	N/A	1:0	“childhood”

Jankovic et al. REF 4	Chart review	1991	USA, North America	cervical radiculopathy & myelopathy, selective cervical rhizotomy*	96/300	49.7	32%	N/A	41.9

Waterson et al. REF 17	Case series	1989	United Kingdom, Europe	cervical myelopathy, laminectomy at C1, posterior fusion at C4, anterior fusion at C3–C4, and forward subluxation of C2–C3 & C3–C4	2/2	61 and 19	N/A	2:0	N/A


Values are shown as mean if exact number was not available in the article. Number of subjects, n; Male, M; Female, F. *Cervical level not indicated in paper.

## Discussion

In this single-center observational study, we found that a diagnosis of significant cervical spine disease following the diagnosis and treatment of CD with BoNT therapy was observed in 5.3% of cases. We found cervical spine stenosis as the most common diagnosis, followed by cervical radiculopathy. Cervical spine complications tended to arise early in the clinical course (<5 years), likely during a period when BoNT therapy had not been optimized. While all individuals required opioid therapy, many patients required additional epidural steroid injections and/or medial branch blocks or surgical procedures such as discectomy, laminectomy and ACDF for controlling symptoms.

Although repetitive twisting and turning of the neck in CD is bothersome and can potentially increase the risk for developing significant cervical spine disease, there is limited literature to support this hypothesis, particularly in the context of BoNT therapy. Published estimates from limited retrospective analysis reveal a range of 18 to 41%. In one of the largest cohorts, Jankovic et al reported that 32% of 300 patients were noted to carry a secondary diagnosis of cervical radiculopathy [4]. The reported spectrum ranged from mild to severe cervical spine involvement, with multilevel pathology commonly observed. Complications have been attributed to increased mechanical stress and accelerated spinal degeneration. In contrast, we observed a lower prevalence of complications (5.3%) attributable to many reasons. Although BoNT was first reported as an effective treatment for CD in 1987, it was not widely accessible during the 1990s and early 2000s for routine clinical use and had not yet been adopted as the first-line therapy. Based on our data analysis, we postulate that the use of BoNT particularly early in the course could potentially reduce the risk of cervical spine complications. Other reasons for a relatively lower prevalence of cervical spine disease could be that during the 1990s, there was limited access to MRI, and this may have hindered early detection and management of cervical spine pathology. Finally, selection bias may have influenced our findings, as the data were derived from patients who were generally satisfied with BoNT therapy (nearly two-thirds reported satisfaction) and therefore continued follow-up in our clinic for at least five years.

We also examined the affected cervical spine levels. Typically, spondylosis changes occur between C4 and C7 with facet joint degeneration as most commonly occurring at C3/4, C4/5, and C5/6 levels [18]. In the context of CD, the continuous abnormal and complex head movements may place greater strain on the upper cervical spine, particularly the C1/C2 articulation, than on the lower segments, where motion is more restricted [18]. Chawda et al found that 14 of 34 CD patients showed moderate to severe degenerative changes, also primarily at the C2/C3 and C3/C4 levels with changes significantly more likely on the side of the dominant torticollis pull [13]. In keeping with some of the previous reports, we found in our cohort, 64.7% of patients showed degeneration at the C2/C3 or C4 levels. Whether patients have greater component of caput or collis can also determine the spine level impacted. Prolonged abnormal head posturing may lead to more complex and asymmetric joint strain compared to a neutral head position. For instance, lateral flexion of the neck to the right will likely induce rotation of the cervical vertebrae to the right, with the spinous processes shifting to the left. Then with an increase in age there is more rotation in the upper cervical spine segment to compensate for reduced mobility in the lower cervical segments. It is plausible that abnormal head positioning and sustained non-physiological stress on the cervical joints contributes to the development of degenerative changes [18].

We found that CD patients with significant cervical spine pathology tended to require opiates for pain control. Among dystonias, CD has the highest prevalence of pain (~70%), especially in patients with pure-caput presentation or later presentation compared to pure-collis type [19] that can contribute to poor quality of life [20,21]. Pain is typically sharp or burning in the neck and shoulders, often radiating toward the side of head deviation and sometimes the same-side arm [22,23]. About one-third of CD patients report a pulling sensation in the neck, and 10–20% experience headaches [24,25]. Emerging evidence suggests that, in addition to sustained muscle contractions, pain in CD may also arise from abnormal nociceptive processing, impaired descending pain inhibition, and structural or network changes in the basal ganglia, cortex, and beyond [26,27,28]. BoNT is an effective treatment for managing CD [29] and the pain related to CD [30]. Although BoNT has substantive antinociceptive effects, all patients in our cohort received opioids, likely due to concurrent musculoskeletal pain generators. Interestingly, in some patients, BoNT improves posture but not pain; in others, it relieves pain without correcting posture [19,31]. While pain is an important feature in CD, there is not much data surrounding the use of opioids to treat pain in this context. A recent study from the Dystonia Coalition cohort found that 11% of dystonia patients met criteria for substance abuse. Opiate use was significantly higher among CD patients with substance abuse compared to those without. Identified risk factors for opiate use in CD included younger age (<55 years), male sex, and comorbid mood disorders [32].

Another notable finding from our literature review was the historical use of BoNT injections in patients with CD undergoing cervical spine surgery. In these reports, BoNT was administered postoperatively to assist with external immobilization and to improve patient tolerance and comfort with halo vests, with repeat injections provided when prolonged immobilization was required [33]. In some reports, perioperative BoNT injection was extended to patients undergoing rigid anterior or posterior instrumentation without external immobilization, with the goal of reducing dystonic movements postoperatively and thereby facilitating spinal fusion [18]. These studies suggest that the use of BoNT may have a stabilizing effect on the cervical spine. Our study has strengths as it represents one of the largest cohorts to date with a clear description of the timeline between the onset of CD and cervical spine disease. Our study also has limitations, including its retrospective design, lack of generalizability as this was a single site study and lack of imaging data for a detailed characterization. We did not capture detailed treatment characteristics, including BoNT dosing or duration of follow-up, for patients excluded from the final cohort (CD patients who did not have clinically significant cervical spine degeneration). While our study suggests that the use of BoNT therapy appears to be associated with a lower risk of cervical spine complications, it is important to note that our study lacked a longitudinal control cohort of BoNT-naïve CD patients for direct comparison. Such a comparison would be clinically impractical and ethically challenging, given that BoNT is an effective first-line therapy for CD. Nevertheless, future studies should include healthy age-matched populations to better determine differences in the prevalence of clinically significant cervical spine complications.

## Conclusion

To summarize, our study found that significant cervical spine pathology can arise as a complication in patients with CD treated with BoNT albeit at a lower rate than that reported in the limited available prevalence data. Pain management can be challenging in these individuals, as many may require opioids, epidural steroid injections, medial branch blocks, radiofrequency ablation or surgical interventions. These findings highlight the importance of careful monitoring in the longitudinal care of CD patients and thoughtful consideration of long-term opioid therapy. BoNT therapy not only alleviates dystonic symptoms and improves quality of life as shown in previous research, however it may potentially reduce the risk of cervical spine complications by stabilizing abnormal mechanical forces acting on the cervical spine. Finally, while the current literature suggests a potential association between CD and cervical spine complications (which is mechanistically plausible), the risk has not been conclusively established or definitively proven. Future studies should be conducted to evaluate whether BoNT may reduce complication rates, for example through analyses of Medicare claims data to assess rates of spinal surgery or injection procedures following a diagnosis of CD and treatment with BoNT. Additional approaches could include imaging-based studies to examine changes in structural or neural compression, as well as semi-direct comparisons with historical cohorts using aggregated datasets.

## Financial Disclosure

SS: Nothing to disclose.

SK: Nothing to disclose.

AWS: reports grant support from the NIH R01NS122943 as PI and Ro1 NS121120-01 as a Co-I. She reports past funding from Benign Essential Blepharospasm Research foundation, Dystonia coalition, Dystonia Medical Research foundation, National Organization for Rare Disorders. AWS has received consultant fees from Merz, Jazz and Acadia. She is the current Vice President for the Tremor Research Group and recent advisor for Supernus and Biogen-Sage.
